# Formulation and Evaluation of Extended-Release Solid Dispersion of Metformin Hydrochloride

**DOI:** 10.4103/0975-1483.63147

**Published:** 2010

**Authors:** SA Patil, BS Kuchekar, AR Chabukswar, SC Jagdale

**Affiliations:** *Department of Pharmaceutics, MAEER’s Maharashtra Institute of Pharmacy, Pune - 411 038, Maharashtra, India*

**Keywords:** Cogrinding, metformin, methocel, solid dispersion, solvent evaporation

## Abstract

The purpose of this research was to formulate and characterize solid dispersion (SD) of metformin hydrochloride using methocel K100M as the carrier by the solvent evaporation and cogrinding method. The influence of drug polymer ratio on drug release was studied by dissolution tests. Characterization was performed by fourier transform spectroscopy (FTIR), ultraviolet, differential scanning calorimetry and X-ray powder diffractometry. The optimized formulation was subjected to accelerated stability testing as per ICH guidelines. Release data were examined kinetically. SD with 1:4 and 1:5 ratio of drug to polymer obtained by solvent evaporation and cogrinding were selected as the best candidates suitable for prolonged-release oral dosage form of metformin.

## INTRODUCTION

Metformin hydrochloride (MET) is an oral antihyperglycemic agent, highly water-soluble, whose low bioavailability and short and variable biological half-life (1.5–4.5 h) needs frequent administrations to maintain effective plasma concentrations.[1] Unlike other biguanide drugs, metformin does not induce lactic acidosis. However, current metformin therapy is suboptimal as it is associated with a high incidence of gastrointestinal side effects, seen in about 30% of the patients, thus making the development of sustained-release forms desirable.[2] An obstacle to more successful use of metformin therapy is the high incidence of concomitant gastrointestinal symptoms, such as abdominal discomfort, nausea and diarrhea, which especially occur during the initial weeks of treatment. Side effects and the need for administration two or three times per day when larger doses are required can decrease patient compliance. Sustained release (SR) formulation that would maintain plasma levels of drug for 8–12 h might be sufficient for once daily dosing for metformin. SR products are needed for metformin to prolong its duration of action and to improve patient compliance.[3]

Sustained or controlled drug delivery occurs while embedded within a polymer that may be natural or semisynthetic or synthetic in nature. The polymer is judiciously combined with the drug or other active ingredients in such a way that the active agent is released from the material in a predetermined fashion and released the drug at a constant rate for a desired time period.[4]

Solid dispersion (SD), in which compounds are dispersed into water-soluble carriers, has been generally used to improve the dissolution properties and the bioavailability of drugs that are poorly soluble in water.[5] SD has also been applied for the controlled release of drugs. Previous reports have shown that by using SDs containing a polymer blend, such as hydroxypropylcellulose (HPMC) and ethylcellulose, it is possible to precisely control the rate of release of an extremely water-soluble drug, such as oxprenolol hydrochloride.[6] SDs of methylcellulose and carboxyvinylpolymer for phenacetin[7] and Eudragit for diclofenac sodium.[8] These studies have shown that there is a linear relationship between the rate of release of the water-insoluble drug and its interaction with the polymer.

A wide array of polymers has been employed as drug-retarding agents, each of which presents a different approach to the matrix concept. Polymers that primarily form insoluble or skeleton matrices are considered as the first category of retarding materials. The second class represents hydrophobic and water-insoluble materials, which are potentially erodible, and the third group exhibits hydrophilic properties.[9]

There are three primary mechanisms by which active agents can be released from a delivery system: diffusion, degradation and swelling followed by diffusion. The release of drug from the matrix depends on the nature of the polymer. Methocel K100M is a hydrophilic polymer that becomes hydrated, swollen and facilitates diffusion of the drug.[10] In the present study, an attempt has been made to formulate metformin as SR SD with the addition of release-retarding polymer methocel K100M in different ratios. The effects of polymer loading on drug release were recorded and the release kinetics was evaluated.

## MATERIALS AND METHODS

MET was kindly supplied by Indoco Remedies (Goa, India) and hydroxypropylmethylcellulose (Methocel K100M) was obtained from Colorcon Asia Pvt. Ltd. (Mumbai, India). All other chemicals and solvents were of reagent grade.

### Preparation of physical mixtures

Physical mixtures of MET and methocel in powder form were mixed in mortar and passed through sieve no. 60. The physical mixtures were prepared in the following ratios: MET:HPMC in the ratios of 1:1, 1:2, 1:3, 1:4 and 1:5.[11]

### SDs prepared by the solvent evaporation method

SDs were prepared by dissolving accurately weighed amounts of methocel and MET in ethanol.[12] After complete dissolution, the solvent was left to evaporate in open air for 2 days. Subsequently, the solid mass was ground and passed through sieve no 60. The sieved ground powders were stored at 25°C in a desiccator in a screw-capped glass vial until use.

### SDs prepared by the cogrinding method

MET was triturated with a minimum quantity of ethanol in a glass mortar until it dissolved.[13] The carrier was then added and the suspension was triturated rapidly at room temperature until the solvent evaporated and passed through sieve no 60.

### Evaluation and characterization of SD

#### Drug content and percent yield

Physical mixtures and SDs equivalent to 10 mg of MET prepared were weighed accurately and dissolved in a 100 ml of distilled water.[14] The stock solutions were filtered through a membrane filter (0.45 mm). The solutions were then diluted suitably in distilled water. The drug content was analyzed at 232 nm using a UV spectrophotometer (Varian Cary 100, Australia). Each sample was analyzed in triplicate. The percentage yield of each formulation was also calculated.

#### Spectroscopic studies

Drug–polymer interactions between MET and HPMC was studied by the spectral shift method. Ten milligrams of each drug, carrier and SDs were dissolved in 100 ml double-distilled water (DDW), filtered using Whatman filter paper no. 41 and degassed by sonication for 30 min.[15] After appropriate dilutions using DDW, the solutions were scanned at 232 nm with a UV spectrophotometer (Varian Cary 100).

#### Dissolution study

The dissolution studies were performed using a US Pharmacopeia 24 type II dissolution test apparatus (Electrolab TDT-08L, Mumbai, India). The samples equivalent to 10 mg MET were placed in a dissolution vessel containing 900 ml of DDW maintained at 37 ± 0.5°C and stirred at 100 rpm. Five-milliliter samples were collected periodically and replaced with a fresh dissolution medium. After filtration through Whatman filter paper no. 41, the concentration of metformin was determined spectrophotometrically at 232 nm.[16] Data were analyzed using PCP Disso software (version 3.0).

#### Dissolution efficiency (DE)

The DE of various SDs was calculated. DE is used as the criterion for comparing the effect of polymer concentration on the release rate. DE is defined as the area under the dissolution curve up to the time “t,” expressed as percentage of the area of rectangle described by 100% dissolution in the same time as in equation 1.[17]

---Eq. 1 DE=∫TY x dt0Y100 X T×100%

where Y is the percent drug release as the function of time, t, T is the total time of drug release and Y_100_ is 100% drug release.

#### Release experiments

In order to gain insight into the drug release mechanism from the SD, release data of selected formulations were examined according to the zero-order, first-order and Higuchi’s square root of time mathematical models, Hixson and Crowell powder dissolution method and Korsmeyer and Peppas model and the release exponent n was calculated.[18] The equations for the said models are given in Table 1. All the dissolution profiles were subjected to model fitting using PCP Disso software (version 3.0).

**Table 1:** Kinetics of optimized solid dispersions of metformin hydrochloride

Model	Equation	SM4		SM5		CM5	
		R^2^	k	R^2^	k	R^2^	k
Zero order	F = k X t (where F is the fraction of drug release, k is the release constant and t is the time)	0.7801	11.7440	0.8382	11.2127	0.5922	11.3547
First order	ln F = k X t (where F is the fraction of drug release, k is the release constant and t is the time)	0.9932	-0.2868	0.9948	-0.2488	0.9611	-0.2416
Higuchi matrix	F = k√t	0.9928	31.9689	0.9972	30.3726	0.9643	31.2308
Hixson and Crowell powder dissolution method	F = 100 (1-(1-kt)^3^)	0.9776	-0.0673	0.9809	-0.0611	0.8901	-0.0606
Korsmeyer and Peppas model^a^	F = kt^n^	0.9978	37.3839	0.9967	32.6910	0.9937	42.2406

a, n (diffusional coefficient) for SM4 n = 0.4512, for SM5 n= 0.4597, for CM5 n=0.3369

A n-value 0.5 is considered consistent with a diffusion-controlled release, whereas a value of 1.0 indicates a zero-order release behavior, and intermediate values (0.5 > n > 1.0) are defined as anomalous non-Fickian transport mechanism.[19]

#### Similarity factor (f2) analysis

The *in vitro* release profile of the marketed MET SR tablets, (Glumet XR^®^, Cipla, Solan-Himachal Pradesh, India) was performed under similar conditions as used for *in vitro* release testing of the test product for the release of metformin. The similarity factor between the two formulations was determined using the data obtained from the drug-release studies.[20] The data were analyzed by the formula shown in equation 2.

---Eq. 2 $$\mathrm{f2}\;=\;50\;\log\;\left\{\left[1+\left(1/N\right)\;\Sigma {\left(\mathrm{Ri}\;-\;\mathrm{Ti}\right)}^{2}\right]{\;}^{-0.5}\;X\;100\;\right\}$$

where N = number of time points, Ri and Ti = dissolution of reference and test products at time i. If f2 is greater than 50, it is considered that the two products share similar drug release behaviors.[21]

#### Diffuse reflectance infrared Fourier transform spectroscopy (DRIFTS)

The DRIFTS spectra of pure MET, physical mixtures and SDs were obtained after appropriate background subtraction using an FTIR spectrometer (FTIR-640 IR, Varian, Australia). About 2–3 mg of the sample was mixed with dry potassium bromide and the sample was scanned from 4,000 to 400 cm^-1^.[22]

#### Differential scanning calorimetry (DSC)

The calorimeter used DSC 823e (Mettler Toledo, Switzerland) was equipped with an intracooler, a refrigerated cooling system. Indium standard was used to calibrate the DSC temperature and enthalpy scale. Nitrogen was used to purge gas through the DSC cell at a flow rate of 50 ml/min and 100 ml/min through the cooling unit.[23] The samples (5–10 mg were hermetically sealed in an aluminum pan and heating was carried out at 5°C/min.

#### X-ray powder diffractometry (XRPD)

The powder X-ray diffraction patterns of powdered samples were recorded using a Philips PW-1729 X-ray diffractometer (Philips House, Cambridge-UK). Samples were irradiated with monochromatized CuKα radiation and a graphite monochromator. The samples were analyzed in the 5–50◦ 2θ range at a scan rate of 0.05◦ per second.[24]

#### Stability studies

Stability studies were conducted on MET SDs along with physical mixtures to assess their stability with respect to their physical appearance, drug content, FTIR spectroscopy and drug-release characteristics after storing them at 40°C and relative humidity (RH) 75% for 1 month and at room temperature for 6 months.[25]

## RESULTS

### Drug contents and percent yield

Table 2 summarizes the actual composition and percentage yield along with abbreviations used for the SDs. Because of difficulty in collecting all the solid material from the flask after ethanol evaporation, the production yields of SDs from solvent evaporation ranged between 87.33 ± 0.49 and 94.67 ± 0.55%. However, satisfactory reproducibility of results when repeating the preparation was observed.

**Table 2:** Abbreviations, percentage drug content, yield and dissolution efficiency for the solid dispersions

Method of preparation	Abbreviations	% drug content^a^	% yield^a^	Dissolution efficiency
Physical mixture 1:1	PM1	98.41 ± 0.78	97.50 ± 1.53	-
Physical mixture 1:2	PM2	102.57 ± 1.25	94.93 ± 0.88	-
Physical mixture 1:3	PM3	103.99 ± 0.87	96.46 ± 1.12	-
Physical mixture 1:4	PM4	96.49 ± 0.69	97.44 ± 0.42	-
Physical mixture 1:5	PM5	99.53 ± 1.08	96.79 ± 0.93	-
Solvent evaporation 1:1	SM1	97.22 ± 0.45	94.67 ± 0.55	93.86
Solvent evaporation 1:2	SM2	94.56 ± 0.36	92.90 ± 1.03	91.00
Solvent evaporation 1:3	SM3	97.41 ± 0.22	87.33 ± 0.49	79.70
Solvent evaporation 1:4	SM4	98.72 ± 1.11	87.46 ± 0.77	67.96
Solvent evaporation 1:5	SM5	96.03 ± 0.55	90.0 ± 0.62	63.97
Cogrinding 1:1	CM1	98.27 ± 0.96	99.20 ± 0.52	92.82
Cogrinding 1:2	CM2	100.06 ± 0.75	93.50 ± 0.39	91.81
Cogrinding 1:3	CM3	95.86 ± 0.80	98.0 ± 1.19	88.19
Cogrinding 1:4	CM4	104.27 ± 0.91	95.33 ± 1.07	78.28
Cogrinding 1:5	CM5	97.93 ± 1.02	93.67 ± 0.67	67.59

a, expressed as mean ± (SD) standard deviation, n = 3

The production yields of SDs from cogrinding ranged between 93.50 ± 0.39 and 99.20 ± 0.52%. The amount of drugs determined in each SD was between 94.56 ± 0.36 and 98.72 ± 1.11% for solvent evaporation and 95.86 ± 0.80 and 104.27 ± 0.91% for cogrinding.

### Spectroscopic studies

The UV spectra of MET and SDs were studied. The observed absorbance in case of SD was reduced than that of the pure drug. There is no shift in the λmax of metformin in the presence of methocel. The induced change in absorbance is attributed, primarily, to the weak hydrogen bonding.

### Dissolution study

**Figure 1:**
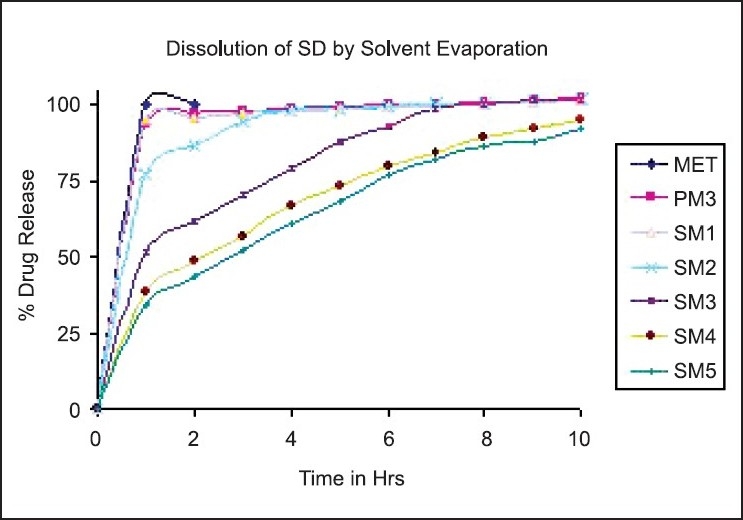
Dissolution profile of metformin hydrochloride, physical mixture and solid dispersions by the solvent evaporation method

**Figure 2:**
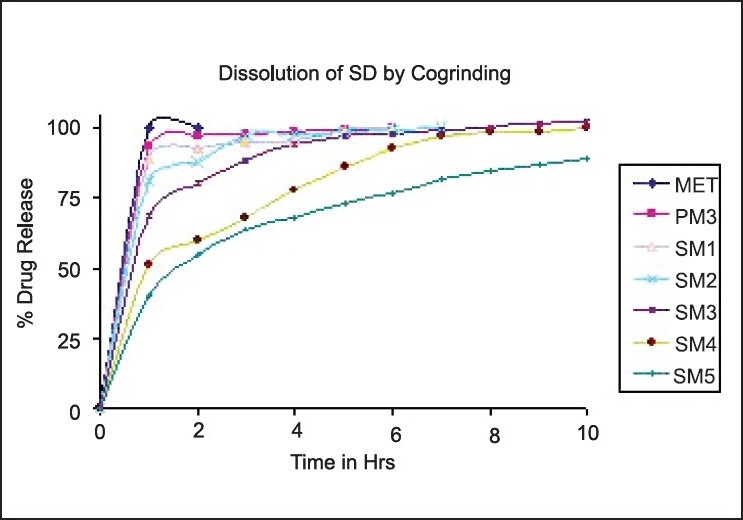
Dissolution profile of metformin hydrochloride, physical mixture and solid dispersions by the cogrinding method

The dissolution profiles of MET SDs along with physical mixture in DDW are shown in Figures 1 and 2 for solvent evaporation and cogrinding, respectively. MET completely dissolved within a few minutes, reflecting its high aqueous solubility. The dissolution from the physical mixture showed approximately the same behavior of pure MET, with only a very slight initial slowing down of the drug dissolution rate due to the presence of the hydrophilic HPMC, which reduces the drug wettability.

### Dissolution efficiency

DE was used to compare the dissolution data, as shown in Table 2. DE values are consistent with the dissolution data. For example, the DE value for the SDs containing 1:1 drug:HPMC ratio is 93.86%, whereas this value decreased to 67.96 and 63.97% for the SDs containing 1:4 and 1:5 ratio of HPMC, respectively, for SDs prepared by the solvent evaporation method.

The SDs showing the desired release profile were selected as optimized batches for further evaluations. The SDs of MET with methocel K100M, which were selected as optimized batches for further evaluation, were SM4, SM5 and CM5, depending on the release profiles as per USP test 2.[26]

### Release experiments

The release data of selected SDs were examined according to the zero-order, first-order and Higuchi’s square root of time mathematical models, Hixson and Crowell powder dissolution method and Korsmeyer and Peppas model and the release exponent n was calculated. Table 1 shows the data for kinetics of SDs. It can be observed that the Higuchi equation was the most suitable mathematical model for describing experimental data only for SM5 containing drug to polymer in a 1:5 ratio, indicating that diffusion through the matrix was the main factor in controlling the drug release rate from SM5 SDs. This was also evidenced by the value of the release exponent n (0.4597), which was rather near to the theoretical one expected for a Fickian diffusion-controlled release mechanism (n = 0.5). Whereas in case of SM4, the Korsmeyer-Peppas equation was most suitable with R^2^ value of 0.9978 and that of first-order equation was 0.9932, indicating that the drug-release pattern is followed by both the equations.

The *in vitro* release pattern of the coground CM5 SDs was analyzed by fitting the dissolution data into various kinetic models. It was observed that the R^2^ value was higher when fitted to Korsmeyer-Peppas equation as compared to zero-order equation, which indicated Peppas as the best fitting kinetic model for CM5.

### Similarity factor (f2)

The similarity factor f2 method can be used to compare two dissolution profiles. Similarity factor analysis between the prepared SDs and the marketed tablet (Glumet XR^®^, Cipla) for the release of MET showed an f2 factor >50; f2 = 50.38, f2 = 59.01 and f2 = 51.57 for SM4, SM5 and CM5, respectively. As shown in Table 3, the f2 factor confirms that the release of MET from the prepared SDs was similar to that of the marketed tablet.

**Table 3:** f2 factor results

Time	Average % release				f2		
	Reference	SM4	SM5	CM5	SM4	SM5	CM5
0	0.00	0.00	0.00	0.00	0.00	0.00	0.00
1	28.30	38.19	33.83	40.09	57.56	69.75	53.81
2	41.68	48.40	43.54	55.08	57.82	72.75	49.25
3	51.92	57.21	52.22	64.03	58.98	75.56	48.28
4	59.17	67.19	60.77	68.23	57.99	77.01	48.93
5	64.78	74.02	68.80	73.12	56.63	75.24	49.60
6	67.34	80.27	77.03	77.26	53.78	66.46	49.67
7	71.17	84.58	82.29	81.65	51.84	61.46	49.56
8	75.90	89.58	86.72	84.60	50.45	58.90	49.88
9	81.35	92.52	88.22	87.13	50.12	58.79	50.62
10	86.81	95.31	92.63	89.46	50.38	59.01	51.57
Average	--	--	--	--	50.38	59.01	51.57

### DRIFTS

**Figure 3:**
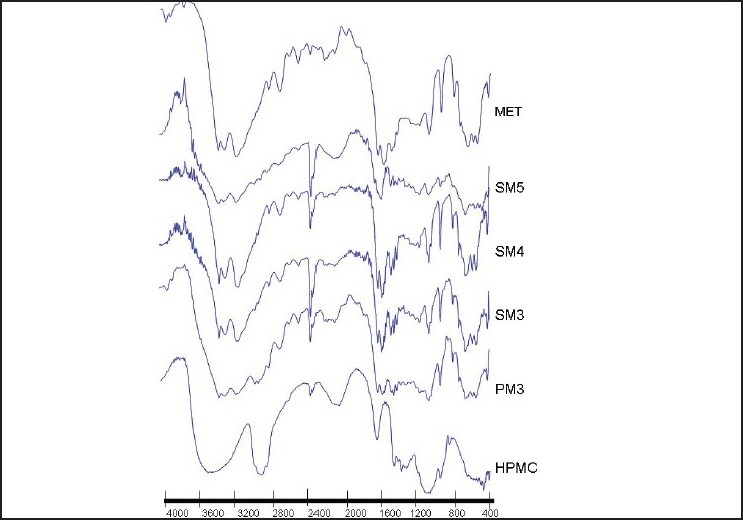
FTIR spectra of metformin hydrochloride, methocel K100M, physical mixtures and solid dispersions by the solvent evaporation method

**Figure 4:**
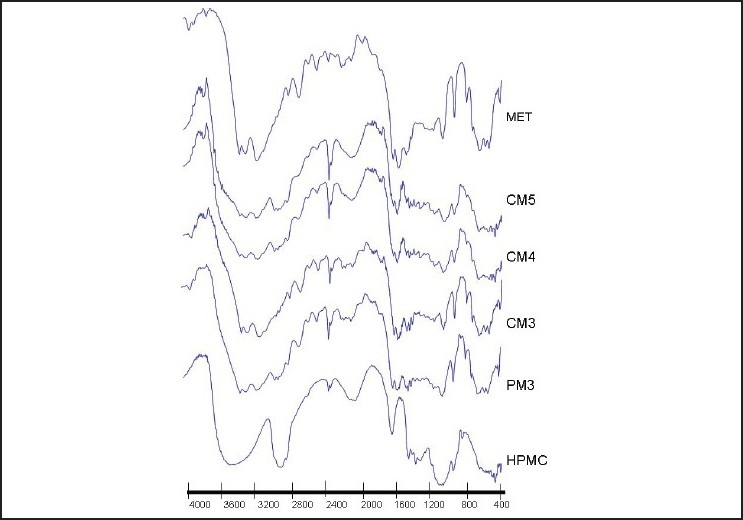
FTIR spectra of metformin hydrochloride, methocel K100M, physical mixtures and solid dispersions by the cogrinding method

The FTIR spectrum of pure MET showed two typical bands at 3369 cm^-1^ and 3294 cm^-1^ relative to the N–H primary stretching vibration and a band at 3155 cm^-1^ due to the N– secondary stretching, and characteristic bands at 1626 cm^-1^ and 1567 cm^-1^, assigned to C-N stretching. IR spectra of HPMC shows typical bands at 3451 cm^-1^ due to O-H stretching, 1644 cm^-1^ due to sugar ring and 1064 cm^-1^ due to C-O (etheric) stretching. The physical mixture spectrum [Figures 3 and 4] can be considered as the sum of pure MET and HPMC spectra. The absolute zero changes of the peak shift in case of physical mixture observed as subtraction of HPMC spectra from PM spectra revealed the pure MET spectra.

### DSC

**Figure 5:**
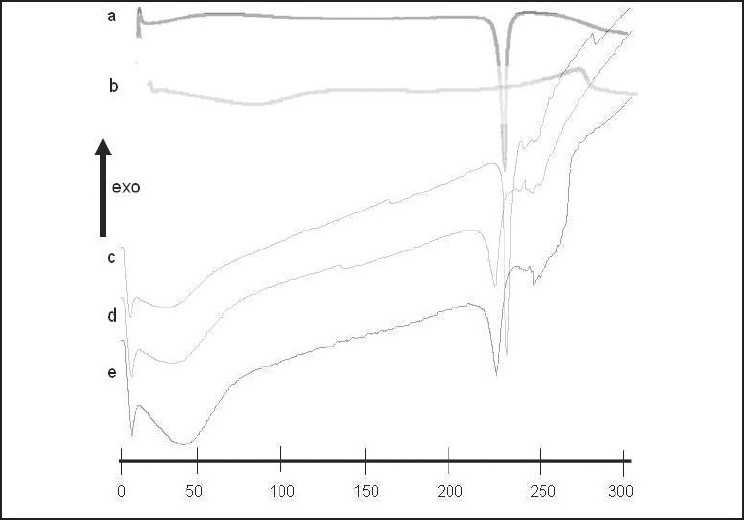
Differential scanning calorimetry curves of (a) metformin hydrochloride, (b) methocel, (c) physical mixtures, (d) SM4, (e) CM5 SD

The thermal curves of MET and of selected SDs along with physical mixture are shown in Figure 5 The thermal curve of pure MET [Figure 5a] exhibited an initial flat profile followed by a sharp endothermic effect, with a T_onset_ at 229.7°C, a T_peak_ at 231.0°C and an associated fusion enthalpy of 292.2 J/g, indicative of its anhydrous crystalline state. The DSC profile of HPMC [Figure 5b] was typical of amorphous substances, showing a large dehydration band in the 50°–120°C temperature range. The thermal curve of the physical mixture [Figure 5c] was practically the sum of those of pure components, showing endothermic effect due to polymer dehydration followed by sharp endothermic peak at 231°C corresponding to the melting point of the drug. The thermograph of SM4 [Figure 5d] and CM5 [Figure 5e] displayed a slight reduction of fusion enthalpy and also endothermic peak, changing the melting point of MET to 226°C.

### PXRD

**Figure 6:**
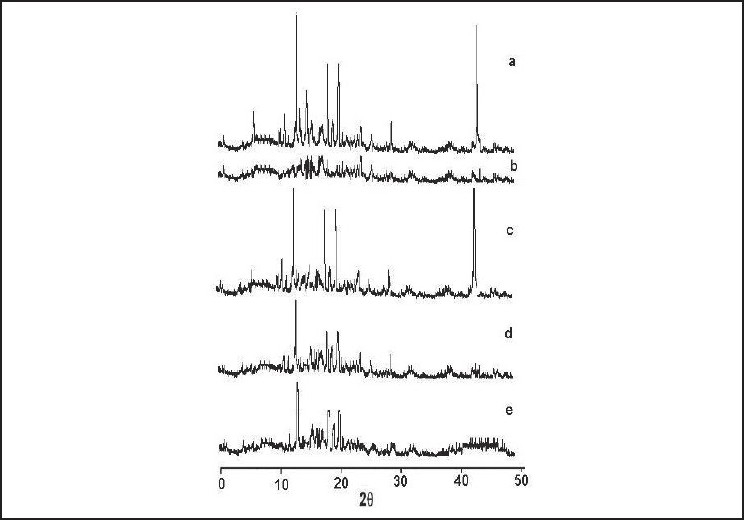
X-ray diffraction pattern of (a) metformin hydrochloride, (b) methocel, (c) physical mixtures, (d) SM4, (e) CM5 SD

Figure 6 shows the powder XRD patterns of pure drug, methocel K100M, physical mixture (1:3), SDs prepared by solvent evaporation (SM4) and SD prepared by the cogrinding method (CM5) with methocel K100M.

In PXRD, sharper diffraction peaks indicate more crystalline material. The sharp, intense representative peaks of pure MET [Figure 6a], notably at 2θ angles, were 17°, 22°, 23°, 31° and 45°. This series of sharp and intense diffraction peaks indicated the crystalline state of pure MET. The diffraction peaks obtained in case of methocel [Figure 6b] is not distinct due to the amorphous nature of the polymer. The diffraction pattern of the physical mixture [Figure 6c] was simply the superimposition of those of pure components. In case of SD prepared by the solvent evaporation method [Figure 6d] and co-grinding method [Figure 6e], the diffraction patterns were much similar to that of physical mixture showing all the intense peaks except at 45° of MET.

### Stability studies

No visible changes in the appearance of the SD were observed at the end of the storage period. Drug content and dissolution of metformin was almost similar to that at time zero during the whole period of investigation. FTIR spectroscopy studies for stability were also conducted on SDs before and after the storage. FTIR studies concluded that there has not been any new bond formation between the drug and the carriers, as there has not been any new peak in the spectra nor has there been deletion of any existing characteristic peak.

## DISCUSSION

### *In vitro* release study

Drug release was fast from SM1 and SM2 SDs, with 97.78 and 95.02% drug released after 3 h, because it underwent erosion before complete swelling could take place. The overall drug release is affected by the rate of water uptake and the diffusion rate of the drug through the swollen gel. High polymer content results in a greater amount of gel being formed. This gel increases the diffusional path length of the drug. Its viscous nature also affects the diffusion coefficient of the drug. As a result, a reduction in drug release rate is obtained. SM3 SD showed faster dissolution rate in an intermediate time of 2–6 h, which did not match with the reference product. On increasing the quantity of HPMC in 1:4 and 1:5 ratio of drug, prolonged release of the drug was achieved, giving drug release of 95.31 and 92.63%, respectively, after 10 h and in the desired pattern. SM4 and SM5 SDs shows similar drug release pattern suggesting that further increasing the polymer ratio above 1:4 does not significantly reduce the release rate of MET.

The drug release from SDs by cogrinding after the first 2 h were 93.03, 88.13, 80.38, 60.34 and 55.08% for CM1, CM2, CM3, CM4 and CM5, respectively. As the ratio of drug to polymer increases, there is a significant decrease in the drug release from the SDs, reaching 90% of dissolved drug after about 3 and 4 h for CM2 and CM3. As the quantity of polymer increases further, there is also decrease in the amount of drug release from SD, giving an extended release up to 10 h for CM4 and CM5. The slow drug-release form SD, namely CM4 and CM5, can be attributed to the low permeability of the polymer, which posed a significant hindrance to fluid penetration and passive drug diffusion. Almost 100% drug is released after 8 h in all the SDs excluding CM5 SDs. After 8 h, the drug release from CM5 SDs was found to be 84.60%, showing compliance with the desired release profile.

The results indicate that there is reduction in drug release as there is increase in the amount of HPMC. However, it seems that there is no change in the release pattern as the amount of HPMC is increased further. DE values for SDs prepared by the cogrinding method were 92.82, 78.28 and 67.59% for CM1, CM4 and CM5, respectively, suggesting that there is reduction in drug release as there is increase in HPMC amount in SD.

### Kinetic studies

The mechanism of drug release from hydrophilic SD after ingestion is complex, but it is based on diffusion of the drug through, and erosion of, the outer hydrated polymer on the surface of the SD. Typically, when the dispersion is exposed to an aqueous solution or gastrointestinal fluids, the surface of the dispersion is wetted and the polymer hydrates to form a gel layer around the drug. This leads to relaxation and swelling of the polymer, which also contributes to the mechanism of drug release. The core of the dispersion remains essentially dry at this stage. In the case of highly soluble drugs like metformin, this phenomenon may lead to an initial burst release due to the presence of the drug on the surface of the dispersion, which is evident by the dissolution data. The gel layer grows with time as more water permeates into the core of the dispersion, thereby increasing the thickness of the gel layer and providing a diffusion barrier to drug release. Simultaneously, as the outer layer becomes fully hydrated, the polymer chains become completely relaxed and can no longer maintain the integrity of the gel layer, thereby leading to disentanglement and erosion of the surface of the dispersion. Water continues to penetrate toward the core of the dispersion, through the gel layer, until it has been completely eroded.

### FTIR

The representative peaks of pure MET were unaltered in PM. The FTIR spectra of the solvent evaporation [Figure 3] and, even more so, of the cogrinding [Figure 4] SDs presented appreciable shifts and reduction in intensity of the characteristic MET bands at 3369 cm^-1^ and 3294 cm^-1^, which may be due to weak hydrogen bonding between the drug and polymer. The weak hydrogen bonding in case of solvent evaporation was less likely to be stronger than SD by cogrinding. The DSC thermographs of SDs revealed that slight downward shift in the endothermic peak of MET also implicit the interaction of MET with methocel. This may be due to the weak hydrogen bonding among NH, NH2 groups of MET and OH group of the methocel. It also suggests the presence of more or less intense solid-state interactions between the components.

### DSC

Slight reduction of fusion enthalpy and lowering of melting temperature of MET observed in the solvent evaporation and, particularly, in the cogrinding SD, can be ascribed to some drug–polymer interactions occurring during sample preparation. Because there is no complete disappearance of melting peak corresponding to both SDs, it indicates retention of the crystalline nature as a consequence of the absence of strong drug–carrier interactions and/or drug inclusion complexation.

### PXRD

The significant decrease in the representative peak intensity height of SD comparatively with the physical mixture and pure MET clearly revealed the conversion of the crystalline nature of MET to the amorphous form.

### Stability studies

Stability studies of the formulation showed that the SD were stable, with no significant change in the physicochemical characteristics. The improved stability of SD could be due to the hydrogen bonding between the drug and HPMC.

## CONCLUSION

The proposed strategy of simultaneously exploiting the combination of the drug with a hydrophilic polymer such as methocel K100M and its SD was effective in adequately modulating the drug-release rate. Spectroscopic studies reveled the absence of interaction between drug and polymer. Release experiments demonstrate that the SR effects can be obtained by simply varying the relative amounts of the polymer in the dispersion. The actual effectiveness of SDs as extended release dosage forms is strongly dependent on the preparation technique used for obtaining SD. SDs prepared by solvent evaporation using methocel K100M were capable of prolonging the release of metformin for 10 h at 80% concentration and by the cogrinding method at 83% concentration of the polymer. The mechanism of drug release was observed to follow the Korsemeyer-Peppas model for SM4 and CM5 and the Higuchi matrix model for SM5 SDs.
